# Vitamin D receptor attenuate ischemia-reperfusion kidney injury via inhibiting ATF4

**DOI:** 10.1038/s41420-023-01456-4

**Published:** 2023-05-12

**Authors:** Shiqi Tang, Xueqin Wu, Qing Dai, Zhi Li, Shikun Yang, Yan Liu, Bin Yi, Jianwen Wang, Qin Liao, Wei Zhang, Hao Zhang

**Affiliations:** 1 Department of Nephrology, The Third Xiangya Hospital, Central South University, Changsha, Hunan Province China; 2 The Critical Kidney Disease Research Center of Central South University, Changsha, Hunan Province China; 3 Department of Anesthesiology, The Third Xiangya Hospital, Central South University, Changsha, Hunan Province China

**Keywords:** Stress signalling, Transcriptional regulatory elements

## Abstract

Activating transcription factor 4 (ATF4) is one of the key effectors of endoplasmic reticulum stress (ERS), ATF4/CHOP pathway-mediated ERS plays an important role in the progression of acute kidney disease (AKI). We have previously reported that Vitamin D receptor (VDR) exert renoprotection in rodent AKI models. However, whether ATF4, as well as ERS, is involved in the protective effect of VDR in ischemia-reperfusion (I/R) induced AKI is unknown. Herein, we showed that VDR agonist paricalcitol and VDR overexpression alleviated I/R-induced renal injury and cells apoptosis with decreased ATF4 and attenuated ERS, while VDR deletion significantly resulted in further increased ATF4, more drastic ERS and renal injury in I/R mice models. In addition, paricalcitol remarkably reduced Tunicamycin (TM) induced ATF4 and ERS with attenuated renal injury, while VDR deletion aggravated the above changes in TM mice models. Moreover, overexpression of ATF4 partially abolished the effect of paricalcitol against TM-induced ERS and apoptosis, while inhibition of ATF4 enhanced the protective effect of paricalcitol. Bioinformatics analysis indicated potential VDR binding sites on *ATF4* promotor sequence which were further confirmed by ChIP-qPCR and dual-luciferase reporter gene assay. In conclusion, VDR attenuated I/R-induced AKI by suppressing ERS partly via transcriptional regulation of *ATF4*.

## Introduction

Acute kidney disease (AKI) is a common clinically critical syndrome with a high incidence of 10–15% in hospitalized patients and more than 30% in critically ill patients [1]. Even though most patients can fully recover in renal function, long-term observation data suggest that a fairly large number of patients who suffer AKI have a significantly increased risk of developing chronic kidney disease compared with those without AKI [2, 3]. Therefore, the prevention and treatment of AKI is an urgent and significant issue for both researchers and clinicians.

Endoplasmic reticulum stress (ERS) is a stress response of cells to the accumulation of unfolded or misfolded proteins in the lumen of the endoplasmic reticulum [4, 5]. BiP/GRP78 was regarded as marker protein of ERS, wherein, activating transcription factor 4 (ATF4) is one of the major effector protein of ERS, which trans-regulate downstream genes involved in apoptosis, inflammation and oxidative stress by binding to cAMP response elements located in its promoter or enhancer regions [6–8]. At present, a large number of studies have confirmed that ERS plays an important role in the pathogenesis of AKI such as ischemic kidney disease and nephrotoxic kidney injury [5, 9–12]. Studies have shown that ATF4 and C/EBP homologous protein (CHOP) is associated with the induction of AKI biomarkers neutrophil gelatinase-associated lipocalin and kidney injury molecule 1 (KIM-1) [13]. Suppressing ATF4/CHOP-mediated ERS could reduce rhabdomyolysis and cisplatin-induced renal damage [14, 15]. As increasing research showed that ATF4/CHOP plays an important role in the development of AKI, thus, understanding the regulatory mechanisms of ATF4 in ERS-mediated renal injury is of great significance for formulating therapeutic strategies against AKI.

Vitamin D receptor (VDR) is a nuclear transcription factor with a wide range of biological effects and is highly expressed in renal tubular epithelial cells. Vitamin D binds to VDR and enters the nucleus to form VDR–RXR complex, which is involved in the transcriptional regulation of downstream target genes [16]. Our research group has confirmed that VDR deficiency aggravated renal tissue inflammation and fibrosis in STZ-induced diabetic mice [17]. In addition, VDR attenuates inflammation and tubular necrosis in cisplatin and LPS-induced AKI mice [18–20]. However, whether VDR plays a protective role in I/R-induced renal injury and the relationship between ATF4 and VDR remains unknown. In this study, we confirmed that ATF4, a key effector protein of ERS, is involved in I/R-induced AKI and VDR activation plays a protective role in I/R-induced AKI by inhibiting ERS. Mechanically, this effect were partly related to the transcriptional regulation of *ATF4* by VDR.

## Results

### VDR agonist alleviated I/R-induced AKI

**Fig. 1:**
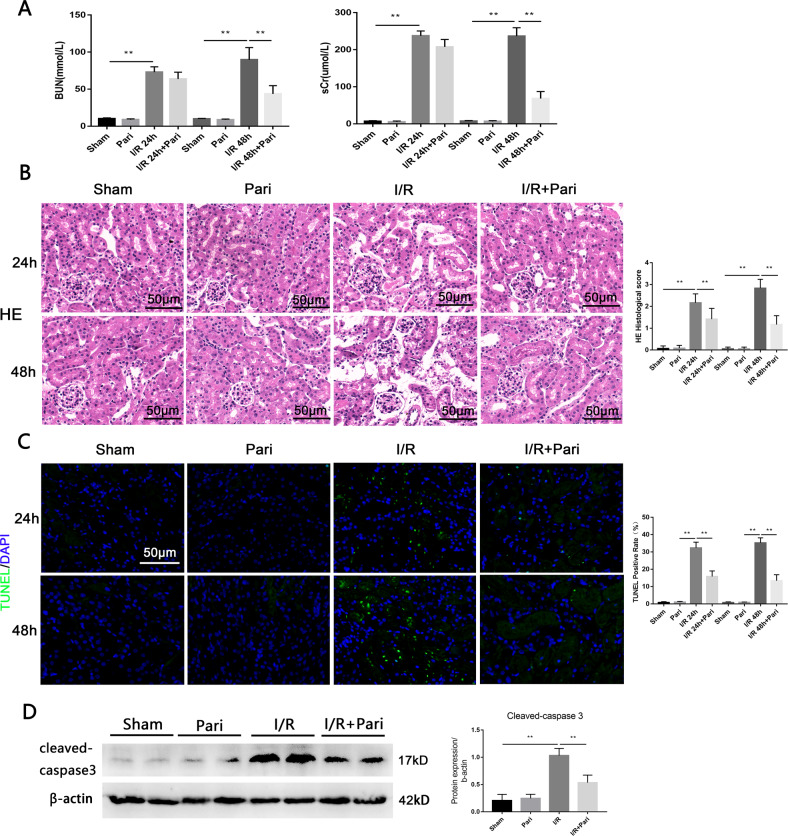
VDR agonist paricalcitol attenuated I/R-induced renal tissue damage and apoptosis. **A** Serum BUN and sCr levels of each group at 24 h or 48 h after I/R. **B** Representative renal images of HE. Scale bar = 50 μm. **C** Representative renal images of TUNEL staining. Scale bar = 50 μm. **D** Western blot and densitometric quantitation of cleaved-caspase3 expression in different groups as indicated. **P < 0.01, *n* = 6 per group.

In AKI model induced by IRI in C57BL/6J mice, our results found that compared with I/R group, pretreatment with VDR agonist paricalcitol significantly decreased urea nitrogen and creatinine levels at 48 h (Fig. 1A). HE staining showed that paricalcitol attenuated I/R-induced tubular injury, including varying degrees of tubular dilation or rupture, tubular cell death, brush border destruction, nuclear nudity, and cell shedding (Fig. 1B). Paricalcitol remarkably reduced cell death induced by IRI as measured by TUNEL (Fig. 1C). In addition, western blot showed that the apoptotic protein cleaved-caspase3 was increased in I/R group, and its elevation was significantly repressed by paricalcitol (Fig. 1D). These data suggested that paricalcitol plays a protective role in IRI induced AKI.

### VDR agonist attenuated I/R-induced ERS

**Fig. 2:**
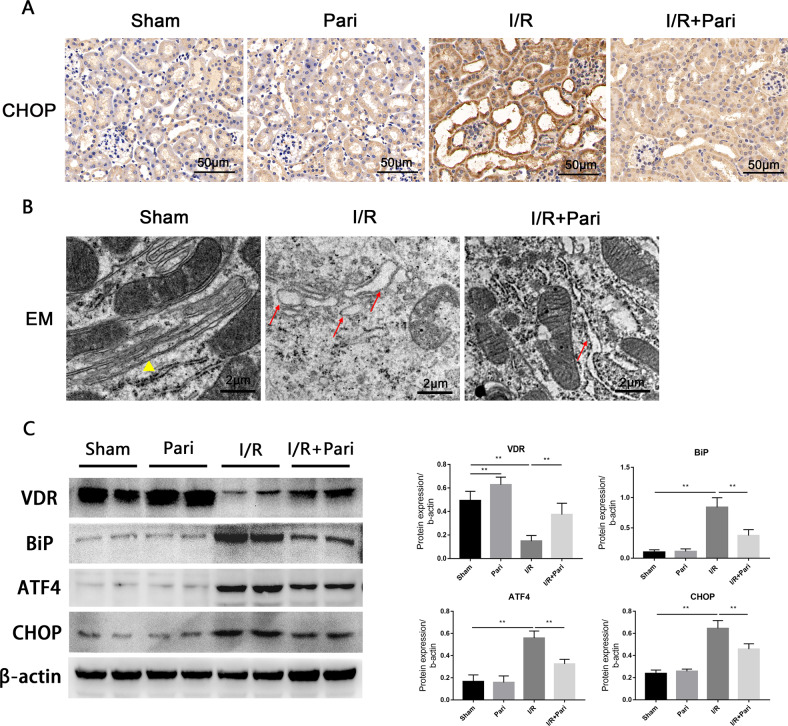
Paricalcitol attenuated I/R-induced ERS. **A** Expression of CHOP was determined by immunohistochemical staining after 24 h of indicated treatment. Scale bar = 50 μm. **B** Representative images of endoplasmic reticulum injury at 24 h under indicated treatment were observed by EM. Triangles represent normal endoplasmic reticulum, arrows represent endoplasmic reticulum changes. Scale bar = 2 μm. **C** Expression of VDR, BiP, ATF4, and CHOP was determined by western blot and densitometric quantitation after 24 h of indicated treatment. **P < 0.01, *n* = 6 per group.

Next, we examined the inhibitory effect of paricalcitol on I/R-induced ERS. The immunohistochemistry results showed that the ERS marker proteins CHOP were significantly elevated in the renal proximal tubular epithelial cells of I/R group and were largely reduced by paricalcitol pretreatment (Fig. 2A). Electron microscope observation showed that the increased endoplasmic reticulum lumen swelling and decreased endoplasmic reticulum area in proximal renal tubular epithelial cells after IRI, which were improved after paricalcitol treatment (Fig. 2B). Furthermore, we found paricalcitol could partly restored VDR expression destroyed by I/R and significantly reduced I/R-induced up-regulation of BiP, ATF4 and CHOP as measured by western blot (Fig. 2C). Taken together, these results suggested that VDR agonist paricalcitol could attenuate I/R-induced ERS, which may through affecting the ATF4/CHOP pathway.

### VDR knockout aggravated I/R-AKI and ERS

**Fig. 3:**
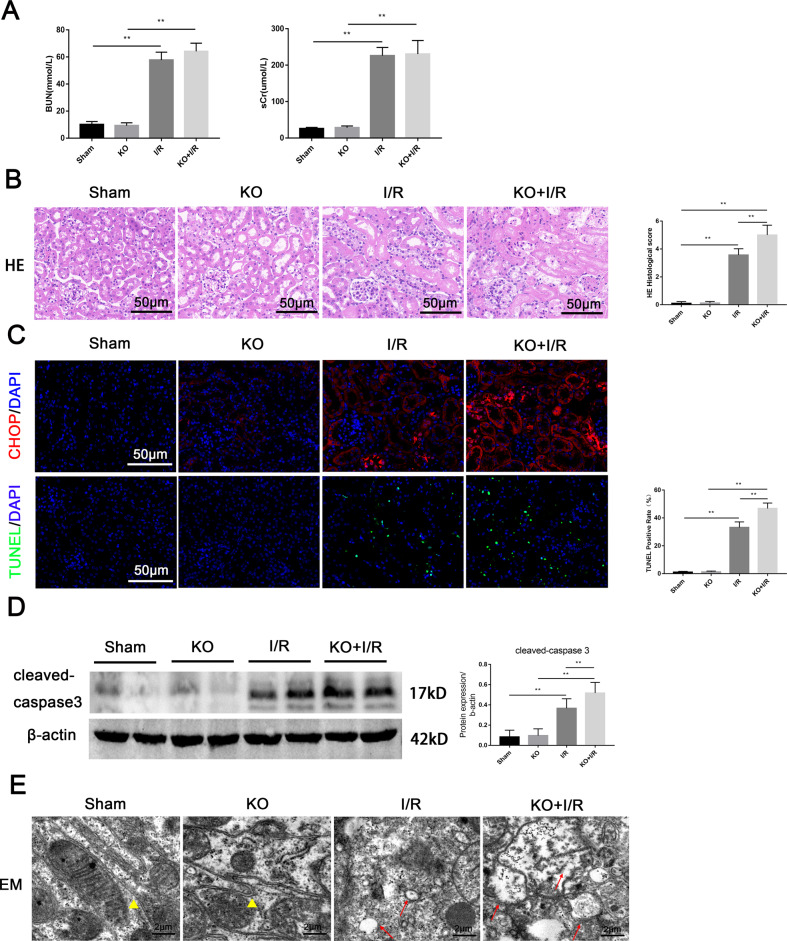
VDR knockout aggravated I/R-induced renal tissue damage and apoptosis. **A** Serum BUN and sCr levels of each group at 24 h. **B** Representative renal images of HE. Scale bar = 50 μm. **C** Representative images of CHOP immunofluorescence staining and TUNEL staining. Scale bar = 50 μm. **D** Expression of cleaved-caspase3 determined by western blot and densitometric quantitation after 24 h of indicated treatment. **E** Representative images of endoplasmic reticulum injury at 24 h under indicated treatment were observed by EM. Triangles represent normal endoplasmic reticulum, arrows represent endoplasmic reticulum changes. Scale bar = 2 μm. **P < 0.01, *n* = 6 per group.

We next induced I/R-AKI on *Vdr*-KO mice to further investigate the regulatory role of VDR on ERS. The results of renal function showed that there were no statistically significant differences in the KO + I/R group compared with the WT-I/R group (Fig. 3A). While deletion of VDR significantly aggravated I/R-induced histological damage (Fig. 3B) as well as CHOP accumulation and cell apoptosis (Fig. 3C, D). Besides, Electron microscopic results showed that VDR knockout aggravated I/R-induced endoplasmic reticulum damage in proximal renal tubular epithelial cells. The endoplasmic reticulum membrane was ruptured, various organelles in the cells were disordered, and normal organelles were hardly seen (Fig. 3E). These results suggested that VDR deficiency aggravated I/R-induced renal injury and ERS.

**Fig. 4:**
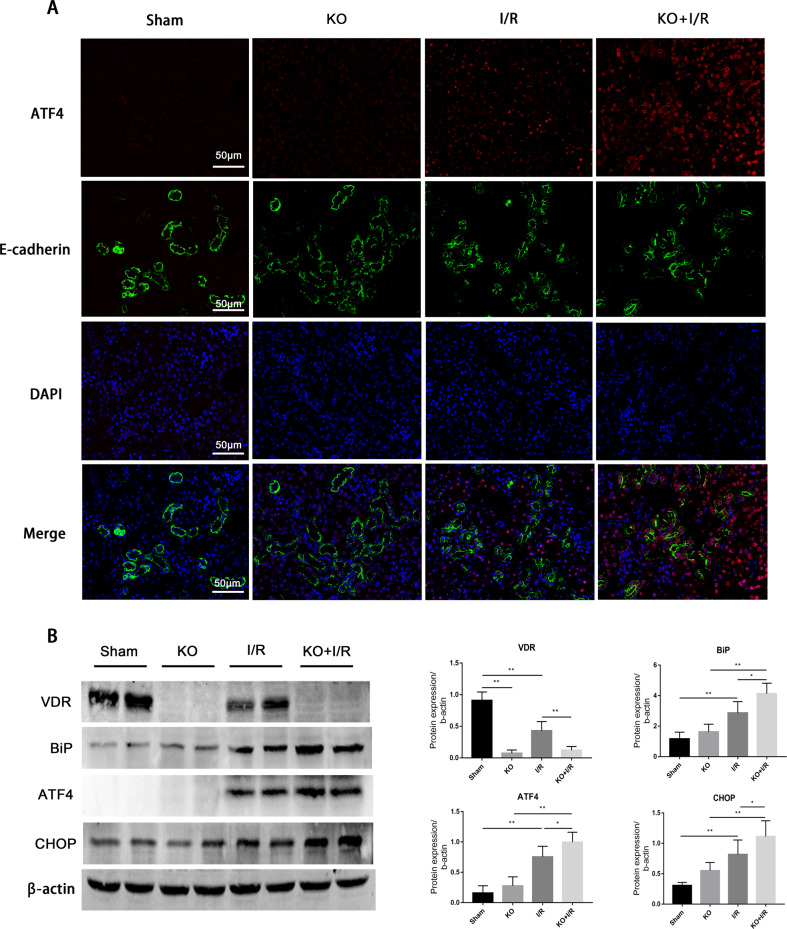
VDR knockout aggravated I/R induced ERS. **A** Representative images of ATF4 immunofluorescence staining (red), green fluorescence represent E-cadherin, blue fluorescence represents nuclear staining. Scale bar = 50 μm. **B** Expression of VDR, BiP, ATF4 and CHOP were determined by western blot and densitometric quantitation at 24 h of indicated treatment. *P < 0.05, **P < 0.01, *n* = 6 per group.

Based on the above-mentioned results that VDR activation may inhibit ERS by affecting ATF4 pathway, we then detected the changes of ATF4 after I/R surgery in *Vdr*-KO mice. The ATF4 belongs to the nuclear transcription factor family which can be expressed both in the cytoplasm and nucleus [21]. The immunofluorescence staining results showed that ATF4 was mainly expressed in renal tubular epithelial cells and mainly concentrated in the nucleus. Compared with the WT mice, knockout of VDR had no effect on the expression of ATF4, while the absence of VDR further promoted the expression of ATF4 after IRI (Fig. 4A). Consistent with the results of immunofluorescence, *Vdr*-KO resulted in more serious ERS and upregulated expression of ATF4 as assessed by western blot (Fig. 4B). Taken together, these results suggested that VDR deficiency aggravated I/R-induced ERS and upregulated expression of ATF4.

### Overexpression of VDR alleviated I/R-AKI and ERS

**Fig. 5:**
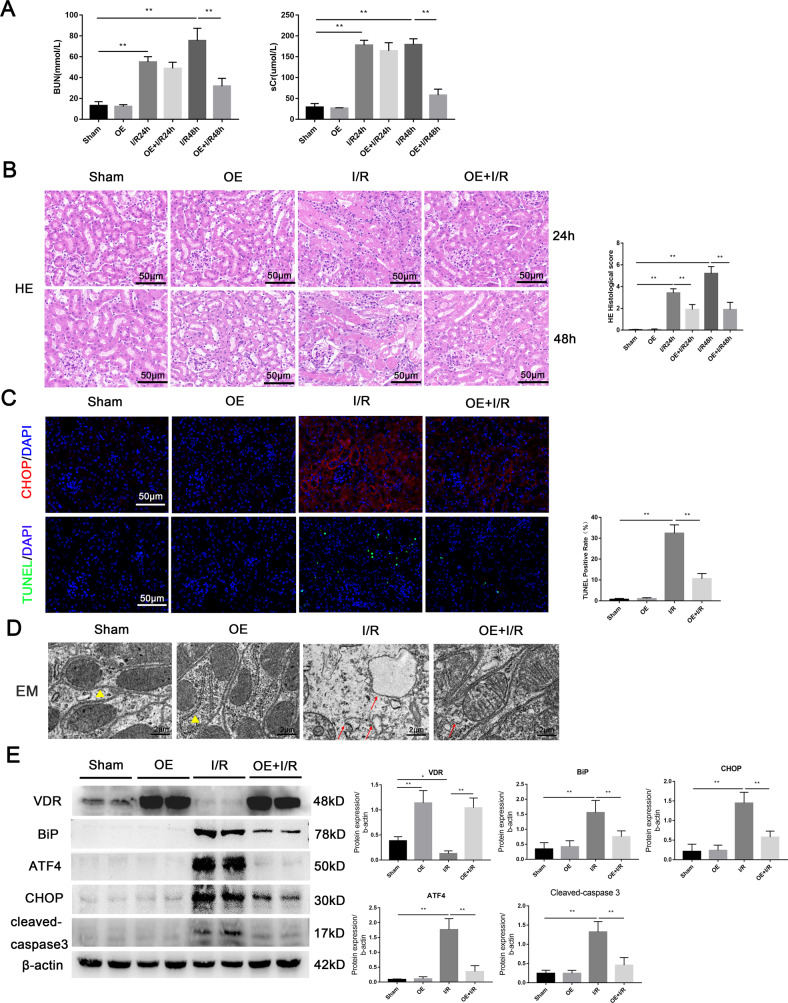
Overexpression of VDR attenuated I/R-induced renal tissue damage, apoptosis, and ERS. **A** Serum BUN and sCr levels of each group at 24 h and 48 h were determined. **B** Representative renal images of HE. Scale bar = 50 μm. **C** Representative images of CHOP immunofluorescence staining and TUNEL staining. Scale bar = 50 μm. **D** Representative images of endoplasmic reticulum injury at 24 h under indicated treatment were observed by EM. Triangles represent normal endoplasmic reticulum, arrows represent endoplasmic reticulum changes. Scale bar = 2 μm. **E** Expression of VDR, BiP, ATF4, CHOP, and cleaved-caspase3 were determined by western blot and densitometric quantitation after 24 h of indicated treatment. *P < 0.05, **P < 0.01, *n* = 6 per group.

We also performed the I/R-AKI model in *Vdr*-OE mice. Our results showed that VDR overexpression significantly ameliorated renal function (Fig. 5A) and histological damage (Fig. 5B). Meanwhile, VDR overexpression reduced I/R-induced CHOP accumulation in renal tubular epithelial cells and reduced cell apoptosis (Fig. 5C). In addition, the results of electron microscopy suggested that VDR overexpression alleviated endoplasmic reticulum damage (Fig. 5D). As expected, VDR overexpression downregulated the expression of BiP, ATF4, CHOP and cleaved-caspase3 (Fig. 5E). These results, on the other hand, strongly suggested that the overexpression of VDR could alleviate ERS and renal injury after IRI, and the inhibition of ERS may be related to the downregulation of ATF4.

### VDR agonist alleviated ERS in tunicamycin-induced mice AKI model

**Fig. 6:**
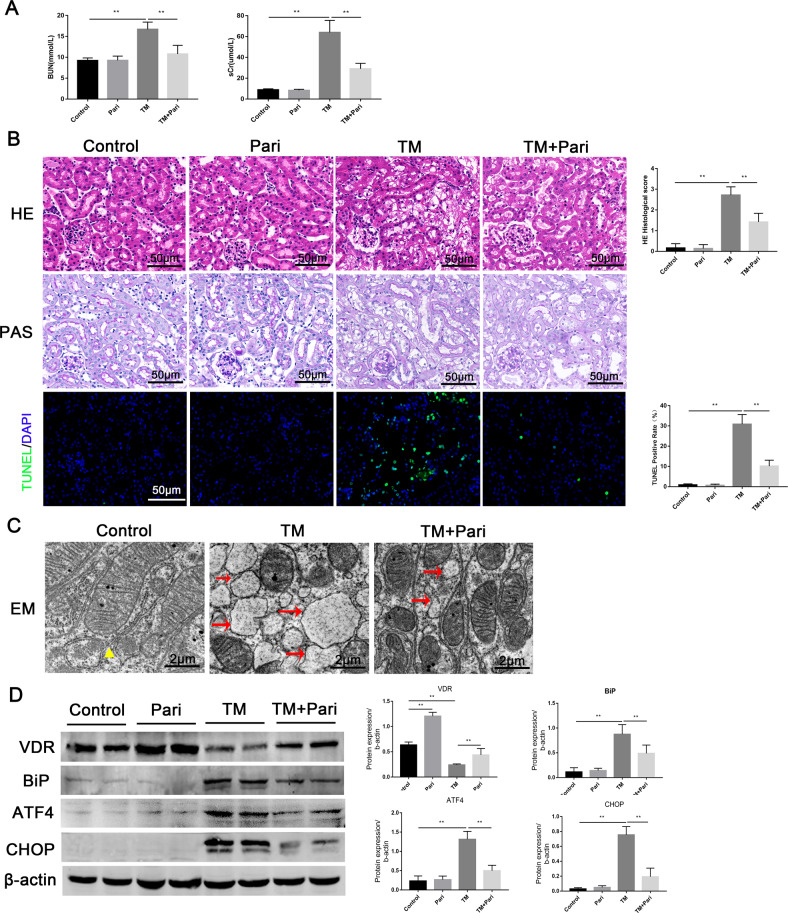
Paricalcitol attenuated TM-induced renal tissue damage, apoptosis, and ERS. **A** Serum BUN and sCr levels of each group at 72 h were determined. **B** Representative HE, PAS, and TUNEL staining (green) images of each group at 72 h. Scale bar = 50 μm. **C** Representative images of endoplasmic reticulum injury at 24 h under indicated treatment were observed by EM. Triangles represent normal endoplasmic reticulum, arrows represent endoplasmic reticulum changes. Scale bar = 2 μm. **D** Expression of VDR, BiP, ATF4, and CHOP was determined by western blot and densitometric quantitation after 24 h of indicated treatment. **P < 0.01, *n* = 6 per group.

To further investigate the inhibitory effect of VDR on ERS, we used an established renal ERS model by using tunicamycin (TM, a classic inducer of ERS). Our results showed that pretreatment with paricalcitol significantly improved renal function as assessed by BUN and sCr (Fig. 6A). Hematoxylin-eosin (HE) and Periodic Acid-Schiff (PAS) staining showed that TM-induced renal tissue injury was characterized by tubular atrophy, loss of brush border, and vacuolation of proximal tubular epithelial cells which were significantly alleviated by paricalcitol. Meanwhile, paricalcitol reduced cell apoptosis induced by TM as detected by TUNEL (Fig. 6B). In addition, TM induced morphological alteration of endoplasmic reticulum injury, as assessed by EM and characterized by the endoplasmic reticulum cavity swollen as oval, were also alleviated by paricalcitol (Fig. 6C). Furthermore, paricalcitol could partly restore VDR down-regulation in TM-induced AKI model and inhibited TM-induced accumulation of BiP, ATF4 and CHOP (Fig. 6D). Together, these data directly showed that VDR activation alleviated ERS and the mechanism may be related to the inhibition of ATF4/CHOP pathway.

In addition, we also performed TM induced-AKI model in *Vdr*-KO mice. Our results showed that *Vdr*-KO aggravated TM-induced renal function (Fig. S1A) and histological damage (Fig. S1B). Electron microscopy results suggested that *Vdr*-KO resulted in more severe endoplasmic reticulum damage (Fig. S1C). Furthermore, *Vdr*-KO aggravated TM-induced ERS and upregulated expression of ATF4 (Fig. S1D). In summarize, these above results provided more direct evidence that VDR knockout aggravated ERS and lead to more severe kidney damage.

### VDR inhibited ERS through the inhibition of ATF4 in renal tubular cells

**Fig. 7:**
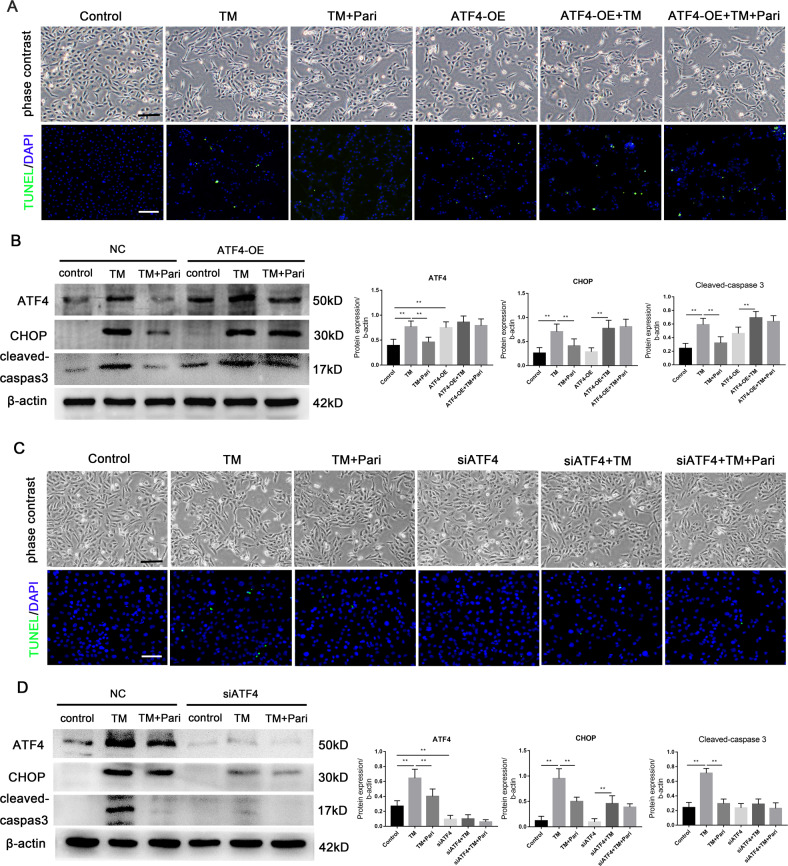
VDR inhibited ERS through the inhibition of ATF4. **A** Representative images of phase contrast microscopy and TUNEL staining. Scale bar ×100. **B** Expression of ATF4, CHOP, and cleaved-caspase3 was determined by western blot and densitometric quantitation after indicated treatment. **C** Representative images of phase contrast microscopy and TUNEL staining. Scale bar ×100. **D** Expression of ATF4, CHOP, and cleaved-caspase3 was determined by western blot and densitometric quantitation after indicated treatment. **P < 0.01, *n* = 3.

To verify whether the effect of VDR on inhibiting ERS is dependent on the regulation of ATF4, we first transfected the ATF4 overexpression plasmid into HK-2 cells. The in vitro study showed that the cells in the TM group displayed a spindle-shaped, fibroblast-like morphology as compared to the cobblestone morphology of the control group. Moreover, TM stimulation resulted in increased cell apoptosis as detected by TUNEL, while these changes were alleviated by paricalcitol. ATF4 overexpression resulted in decreased cell number and increased apoptosis compared to the control group, thus weakening the protective effect of paricalcitol on cells (Fig. 7A). Furthermore, paricalcitol significantly reduced TM-induced ERS and apoptosis, which were largely abolished by ATF4 overexpression as detected by western blot (Fig. 7B). In addition, we also transfected ATF4 siRNA into cells to examine the effect of paricalcitol on TM-induced ERS. Our results showed that the inhibition of ATF4 reduced TM-induced cell morphological changes and apoptosis (Fig. 7C) as well as enhanced the protective effect of paricalcitol on TM-induced ERS and apoptosis (Fig. 7D). Together, the above results showed that VDR inhibited ERS and reduced apoptosis partially by downregulating ATF4.

### ATF4 was transcriptionally regulated by VDR

**Fig. 8:**
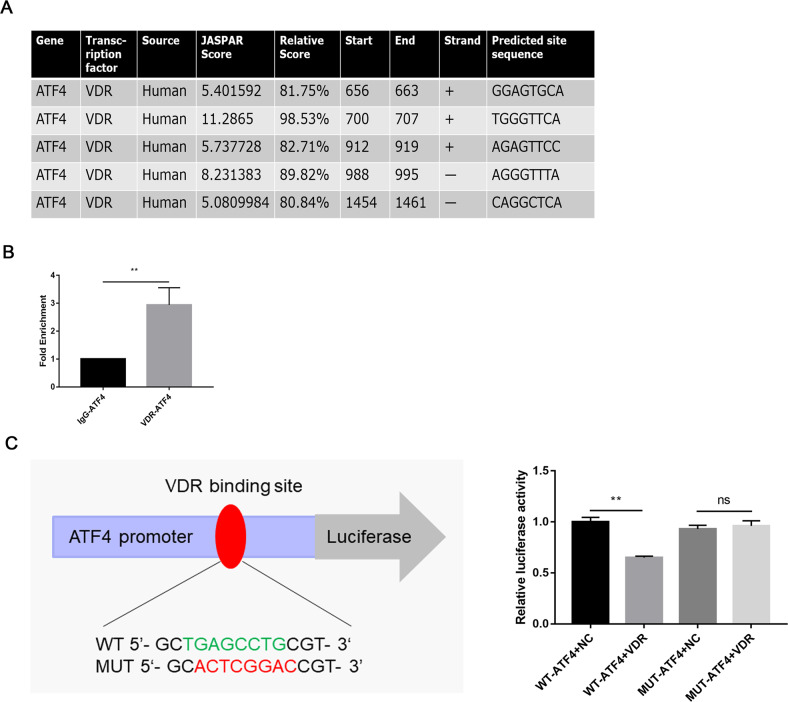
ATF4 was transcriptionally regulated by VDR. **A** VDR binding sites in the promoter region of human ATF4 gene predicted by JASPAR online analysis (http://jaspar.genereg.net). **B** Chromatin immunoprecipitation (ChIP) study and **C** dual-luciferase reporter assay were conducted to confirm the interaction between ATF4 gene promoter regions with transcription factor VDR. Data are presented as the mean ± SD, **P < 0.01. *n* = 3.

As VDR is a nuclear transcriptional factor, thus to further illustrate the regulatory mechanism of VDR on ERS, we continued to explore the intrinsic relationship between VDR and ATF4. By using JASPAR database (http://jaspar.genereg.net), we identified putative VDR-binding sites for ATF4 (Fig. 8A). Then, the ChIP assays was performed to validate these binding sites which revealed the potential binding between the promoter region of VDR and ATF4 in HK-2 cells (Fig. 8B). Subsequently, we determined the interaction between VDR and ATF4 promoter region by luciferase reporter gene assay. We constructed *ATF4* luciferase reporter vectors containing wild-type (WT-*ATF4*) or mutated (MUT-*ATF4*) VDR-binding sequences. The dual-luciferase reporter assay results showed that the luciferase activity of WT-*ATF4* group was significantly decreased after co-transfection with the VDR compared with the MUT-*ATF4* group (Fig. 8C). Collectively, these data indicated direct binding of VDR to the ATF4 promoters, confirming that VDR regulates ATF4 at the transcriptional level.

## Discussion

Our present study has observed that overt ERS was induced in IRI-AKI mice model which is consistent with previous report [10, 22–25]. However, the underlying regulating mechanism of ERS in AKI need to be further elucidated. Both our work and others have demonstrated a destructive effect of ERS in the occurrence and progression of I/R-induced acute kidney injury. Therefore, inhibition of ERS could reduce tissue damage in AKI and be an effective target for the prevention and treatment of AKI. It has been reported that Intermedin (IMD, a novel calcitonin/calcitonin gene-related peptide family) inhibited BiP/GRP78 and CHOP, improved renal function in rats after IRI [26]. Fan, et al. showed that knockdown of renal tubular reticulon-1A (RTN1A) in mice attenuated ERS and apoptosis in nephrotoxic AKI [27]. Pharmacological inhibition of fatty acid-binding protein 4 reduces renal tubular epithelial cell damage induced by rhabdomyolysis via inhibiting ERS [14]. Although animal and cell culture studies have revealed evident beneficial of ERS regulation or inhibition via chemical reagent inhibitor like TUDCA and 4-PBA [5, 28, 29], further application of these reagents were largely limited due to shortness of safety data.

In our present work, we reported that VDR activation via paricalcitol (a clinically approved vitamin D analog) or transgenic overexpression inhibited ERS marker BiP/GRP78, significantly attenuated cells apoptosis, endoplasmic reticulum injury and renal tissue damage in IRI induced AKI mice model. More importantly vitamin D/VDR signaling significantly reduced the expression of ATF4 and its downstream CHOP. Conversely, VDR knockout obviously promoted ERS, aggregated renal injury and further increased ATF4 as well as CHOP in I/R mice model, suggesting that VDR reduced ERS partly through ATF4/CHOP pathways. In addition, we found that paricalcitol also had significant protection in tunicamycin (a ERS inducer) induced AKI model and HK-2 cell injury with inhibited the activation of ATF4/CHOP pathway. While, VDR deletion significantly resulted in further increased ATF4, more drastic ERS and renal injury in TM mice models. Overall, these data has provided more direct evidence that VDR activation could inhibit ERS may through ATF4/CHOP pathway.

As an important effector protein of ERS, ATF4 activates its downstream CHOP involved in ERS-related apoptosis [9, 30]. Studies have shown that CHOP-mediated apoptosis and pyroptosis play a pathogenic role in renal ischemia-reperfusion injury (IRI) [23, 31]. In addition, ATF4 and CHOP triggered protein synthesis that may cause proteotoxicity, supporting the pathogenic role of ATF4/CHOP pathway under ERS [32]. Our research showed that the level of ATF4 is significantly increased in both I/R and TM-induced mouse models, while VDR activation could inhibit ATF4 and downstream CHOP, improve renal function, which further confirmed the importance of ATF4/CHOP pathway in the progress of AKI. Besides, we observed in vitro cell experiments that the inhibitory effect of paricalcitol on ERS was partially abolished by overexpression of ATF4, while silencing ATF4 expression enhanced the protective effect of paricalcitol on ERS, suggesting that the regulation of ERS by VDR at least partly depends on ATF4. Taken together, our work found that VDR activation has a cytoprotective effect in I/R-AKI by inhibiting ERS, which is partly mediated by regulation of ATF4. Meanwhile, the regulatory effect of VDR activation on ATF4 has not been reported before, and these works may indirectly suggest a potential relationship between vitamin D/VDR signaling with ERS.

Additionally, it should be noted that the expression of ATF4 was only slightly upregulated in the kidney tissue of *Vdr*-KO mice in sham control compared with WT mice, which indicates that without evident ischemic stress, VDR deficiency was not enough to induce ERS. As ERS is a dynamic network of the stress response [33], the expression of ATF4 in *Vdr* knockout mice may also be affected by other factors. While after I/R insult, the expression of ATF4 was significantly upregulated in *Vdr*-KO mice compared with WT mice. Combined with the ChIP and dual-luciferase reporter assay results, these data are sufficient to support the trans-regulation of ATF4 by VDR although VDR does not seem to be a key regulator of ATF4. On the other hand, ATF4 may not be the only one regulated by VDR in ERS either. Given that the overall protective effect of VDR on ERS and kidney injury is clearly there, we speculate that VDR may also affect ERS through other mechanisms, such as ATF6 or XBP1 which await further study.

In conclusion, our present work has confirmed the role of ERS in I/R-induced AKI, and VDR activation attenuates I/R-induced AKI by inhibiting ERS, the mechanism of which is related to the transcriptional regulation of *ATF4* by VDR. Targeting ATF4 may provide a novel and promising approach for AKI prevention and treatment, VDR activation may be a potential strategy.

## Materials and methods

### Reagents and antibodies

The HK-2 cells were obtained from the American Type Culture Collection (ATCC, USA). DMEM/F12 was obtained from HyClone (SH30023.01, USA). Fetal bovine serum was obtained from Gibco (FBS-CBT, USA). Tunicamycin (TM) was acquired from Abcam (ab120296, UK). Paricalcitol was obtained from USP (1499414, USA). ATF4 plasmid and siRNA were constructed in Guangzhou Ribo Biotechnology Co, Ltd. lipofectamine3000 was acquired from Invitrogen (L3000-018, Guangzhou, China). The following antibodies were used in the experiments: anti-VDR (Abcam, ab109234), anti-ATF4 (Abcam, ab184909), anti-BiP (Proteintech,11587-1-AP, Wuhan, China), anti-CHOP (Proteintech,15204-1-AP), anti-cleaved-caspase3 (Abcam, ab214430) and anti-β-actin (Proteintech,20536-1-AP).

### Animal studies

C57BL/6J mice were purchased from Slyke jingda Biotechnology Company (Certificate SCXK2016-0002; Hunan, China). VDR knockout (*Vdr*-KO) and VDR specifically overexpressed in kidney proximal tubular epithelial cells (*Vdr*-OE) were constructed in cooperation with the Model Animal Research Center of Nanjing University (Nanjing, China) as reported in previous studies [18, 20]. All animals were randomly grouped according to the principle of randomization, and the control group of gene mice comes from mice born in the same litter. All of them were fed under SPF conditions. C57BL/6J mice, *Vdr*-KO mice and *Vdr*-OE mice were subjected to bilateral renal artery clipping for 30 min and reperfusion for 24 or 48 h. Paricalcitol (0.2 μg/kg) or equal volume of normal saline was intraperitoneally injected for 5 consecutive days before surgery. To investigate the role of VDR in ERS, C57BL/6J, *Vdr*-KO mice were intraperitoneally injected with TM (1 mg/kg body weight) or control vehicle (150 mM of Dextrose) for 24–72 h. This study was reviewed and carried out in central south university department of laboratory Animals. All experiments using animals were reviewed and approved by the Laboratory Animal Ethics Committee of Central South University (CSU-2022-01-0072).

### Cell culture and treatment

HK-2 cells were cultured in DMEM containing 10% fetal bovine serum in F12 (1:1). HK-2 cells were transfected with ATF4 plasmid or siRNA by lipo3000 (Invitrogen, USA). Before transfection, cells were seeded in six-well plates at 5 × 10^4^/well. After incubation at 37 °C for 24 h, cells were transfected with 4 μg/ml plasmid or 50 nM siRNA, mixed with serum-free medium and transfection reagent according to the manufacturer’s instructions for 6 h, then cells were changed to complete medium with or without paricalcitol (200 nM) for 24 h. The protein was extracted after 24 h incubation with or without tunicamycin (3 μg/ml).

### Renal tissue histopathological and TdT-mediated dUTP nick end labeling (TUNEL) assessment

Paraffin-embedded kidney tissues were cut into 3 μm thick sections for H&E staining, PAS staining, or TUNEL fluorescence staining. The degree of renal interstitial injury in mice was assessed as previously reported [34], cell death was quantified.

### Immunohistochemical staining

Tissue sections (4 μm) were deparaffinized and rehydrated, and microwave antigen retrieval was performed using citrate buffer. After cooling, peroxidase activity was blocked with 3% hydrogen peroxide for 10 min at room temperature. Slides were then blocked with 5% BSA for 1 h at room temperature and incubated with primary antibody overnight at 4 °C (CHOP 1:100). After washing with PBS the next day, slides were incubated with secondary antibody for 1 h at room temperature, and finally washed with PBS. The nuclei were counterstained with hematoxylin, and the protein expression was observed by DAB staining.

### Immunofluorescence staining

Tissue sections (4 μm) were deparaffinized, rehydrated, and subjected to microwave antigen retrieval in citrate buffer. After cooling, peroxidase activity was blocked with 3% hydrogen peroxide at room temperature for 10 min, then blocked with 5% BSA at room temperature for 1 h. Next, incubated with primary antibody overnight at 4 °C (ATF4:1:100; CHOP 1:100). The next day, the slides were washed with PBS, incubated with fluorescent secondary antibody for 1 h at room temperature in the dark, then washed with PBS, and finally stained with DAPI.

### Endoplasmic reticulum morphology observation by electron microscope

1 mm^3^ of fresh kidney cortex was quickly placed in electron microscope fixative (Glutaraldehyde, 4%) at 4 °C. Then the tissue was embedded and cut into ultrathin sections of 60–80 nm, followed by uranium-lead double staining. The morphology of endoplasmic reticulum in proximal renal tubular epithelial cells was observed by transmission electron microscope and images were collected.

### Western blot analysis

The extracted tissue proteins were separated from total proteins by SDS/PAGE and electrotransfered to PVDF membranes (Millipore, IPVH00010, USA). Membranes were blocked with 0.1% BSA solution on shaker for 1 h, then incubated with corresponding primary antibody overnight at 4 °C, finally incubated with chemical secondary antibodies for 1 h at room temperature the next day. Membranes were visualized by Image Studio software and band intensities were quantified by Image J analysis software. All experiments were repeated at least three times.

### Chromatin immunoprecipitation (ChIP) assay

Experiments were performed according to the instructions of the ChIP kit (Abcam, ab500). Cells were fixed with formaldehyde and the lysates were sonicated. Samples after ultrasonic treatment were subjected to immunoprecipitation (VDR, CST 12550, USA). After purification, qRT-PCR analysis was performed. Primers were designed according to the VDRE binding sites of ATF4 (forward, AGCCCTCACACTGGAGGTGCA, reverse, CCCGCAGGCGTGCCGTCTC).

### Dual-luciferase reporter gene assay

ATF4 wild-type (WT) and ATF4 mutant (MUT, binding site mutation) sequences were constructed and inserted into pGL3-basic vector. Transfection was performed using Lipofectamine 3000. After 48 h of transfection, luciferase activity was measured by Dual-luciferase reporter assay system (Promega, USA).

### Statistical analysis

Data were analyzed using SPSS 22.0 statistical software and expressed as mean ± standard deviation. Statistical comparisons were performed using Student’s *t* test or one-way analysis of variance as appropriate, post-hoc comparisons used to determine differences for the means where multiple comparison of the means occur. Statistical significance was defined as *p* < 0.05.

## Supplementary information

Figure S1

Supplementary figure legend

Original western blots

## Data Availability

The data that support this study are available from the corresponding author upon reasonable request.
