# Supplementary material for: Vitamin D receptor attenuate ischemia-reperfusion kidney injury via inhibiting ATF4
Source: Cell Death Discov. 2023 May 12;9:158. doi: 10.1038/s41420-023-01456-4 (PMC10182024; doi:10.1038/s41420-023-01456-4)
Supplement: Supplementary file 2 — Supplementary figure legend [file 41420_2023_1456_MOESM2_ESM.docx]

**Fig.S1 VDR knock out aggravated TM induced renal tissue damage and ERS.**

**A** Serum BUN and sCr levels of each group at 72h were determined. **B** Representative images of HE and PAS staining. Scale bar = 50 μm. **C** Representative images of endoplasmic reticulum injury at 24h under indicated treatment were observed by EM. Triangles represent normal endoplasmic reticulum, arrows represent endoplasmic reticulum changes. Scale bar = 2 μm. **D** Expression of BiP, ATF4 and CHOP at 72h were determined by western blot and densitometric quantitation after indicated treatment. **P* < 0.05, ***P* < 0.01, n = 6 per group.
