# Supplementary material for: Vitamin D receptor attenuate ischemia-reperfusion kidney injury via inhibiting ATF4
Source: Cell Death Discov. 2023 May 12;9:158. doi: 10.1038/s41420-023-01456-4 (PMC10182024; doi:10.1038/s41420-023-01456-4)

Fig.1

Cleaved-caspase3 17kD

Sham Pari I/R I/R+Pari

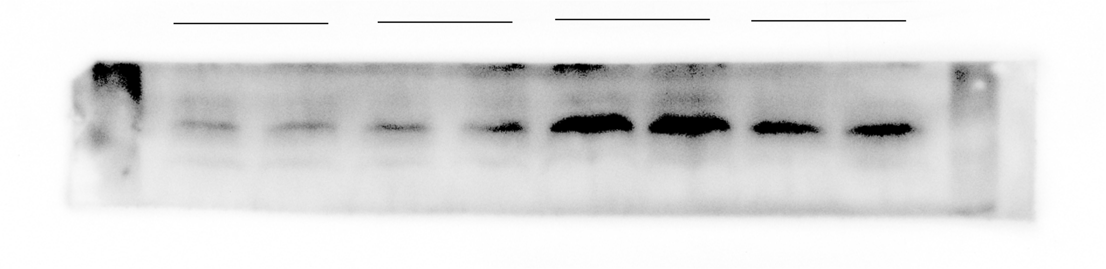

b-actin 42kD

Sham Pari I/R I/R+Pari

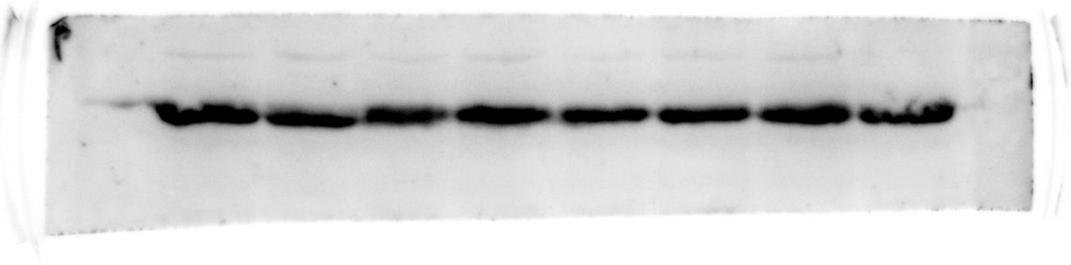

Fig.2

VDR 48kD

Sham Pari I/R I/R+Pari

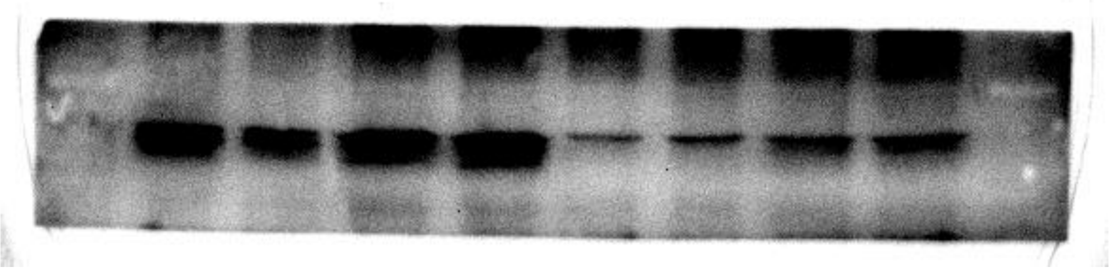

BiP 78kD

Sham Pari I/R I/R+Pari

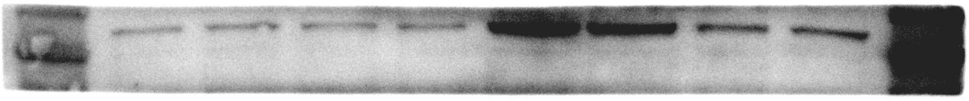

ATF4 50kD

Sham Pari I/R I/R+Pari

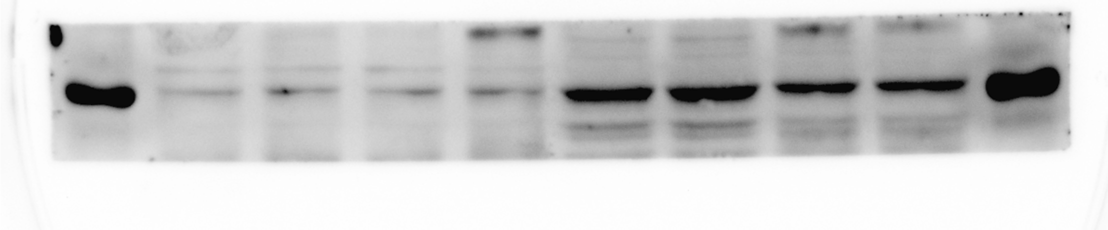

CHOP 30kD

Sham Pari I/R I/R+Pari

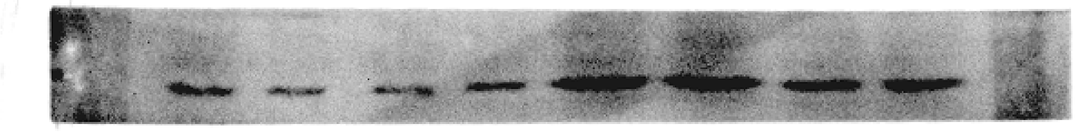

b-actin 42kD

Sham Pari I/R I/R+Pari

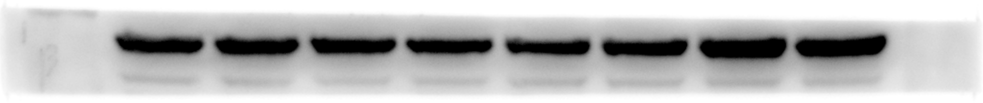

Fig.3

Cleaved-caspase3 17kD

Sham KO I/R KO+I/R

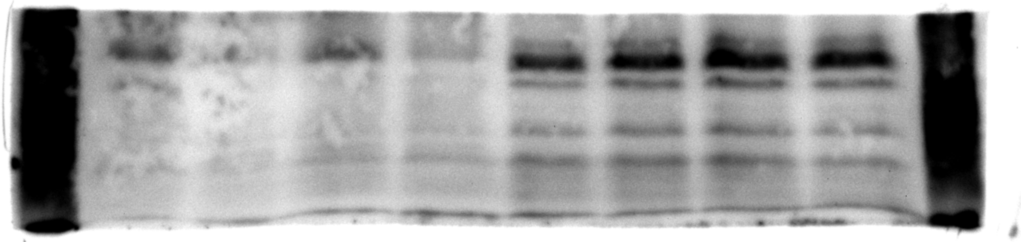

b-actin 42kD

Sham KO I/R KO+I/R

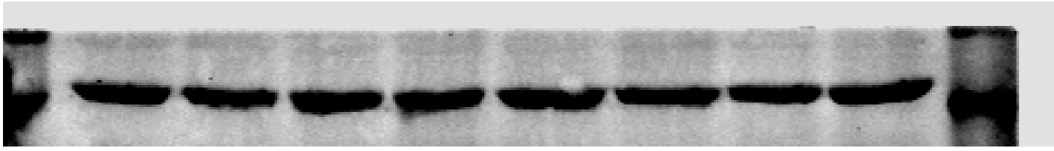

Fig.4

VDR 48kD

Sham KO I/R KO+I/R

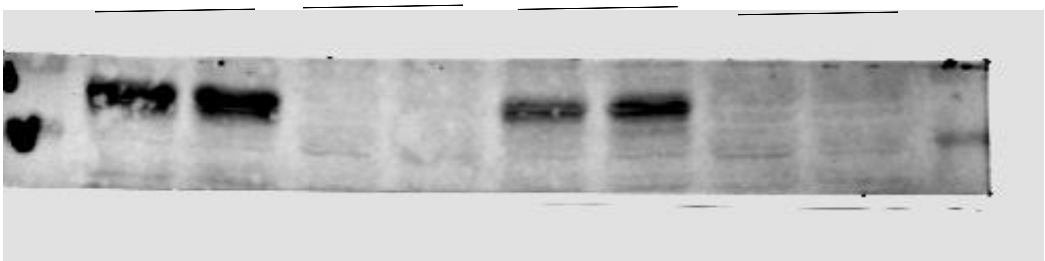

BiP 78kD

Sham KO I/R KO+I/R

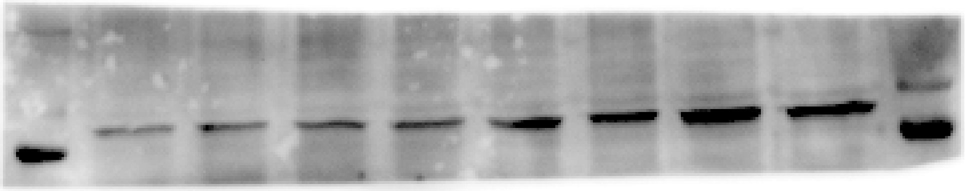

ATF4 50kD

Sham KO I/R KO+I/R

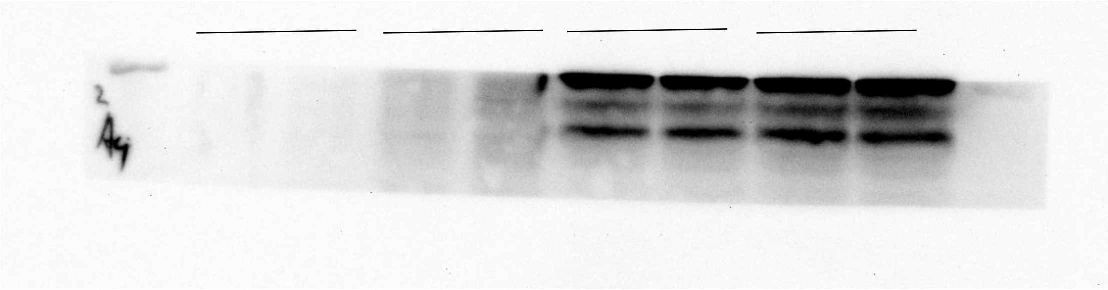

CHOP 30kD

Sham KO I/R KO+I/R

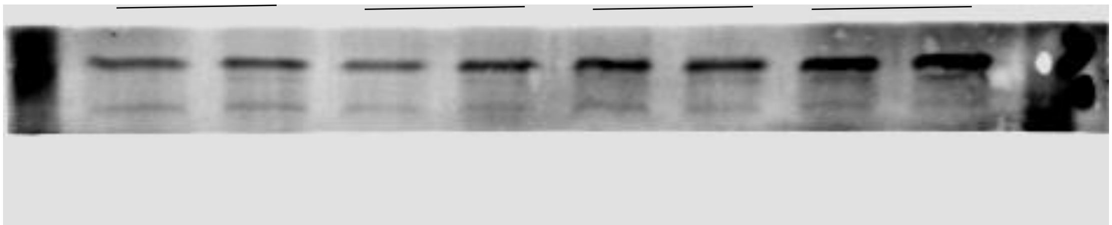

b-actin 42kD

Sham KO I/R KO+I/R

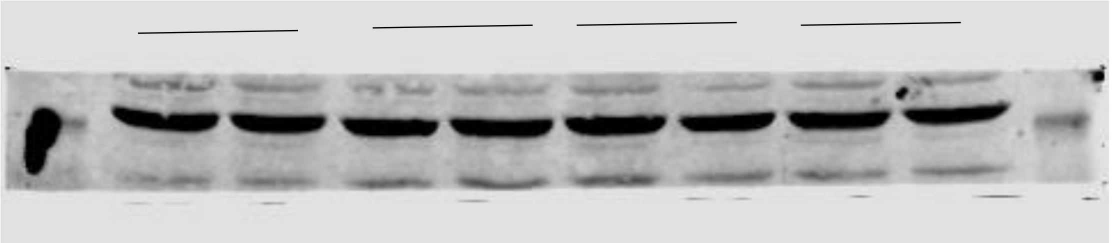

Fig.5

VDR 48kD

Sham OE I/R OE+I/R

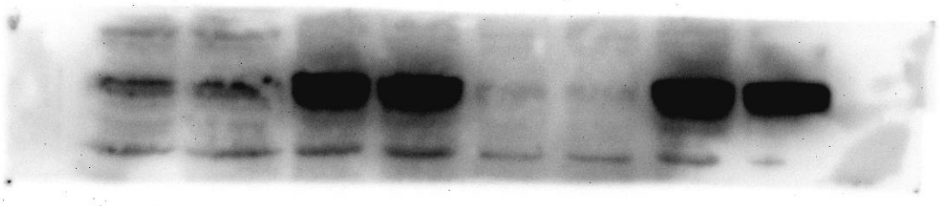

BiP 78kD

Sham OE I/R OE+I/R

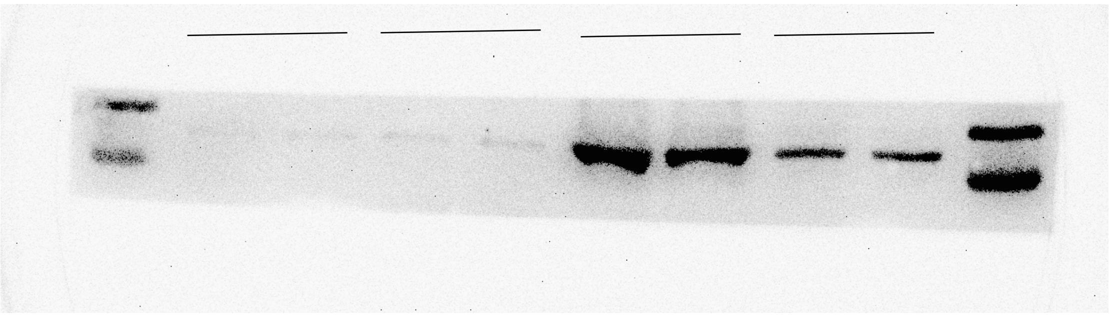

ATF4 50kD

Sham OE I/R OE+I/R

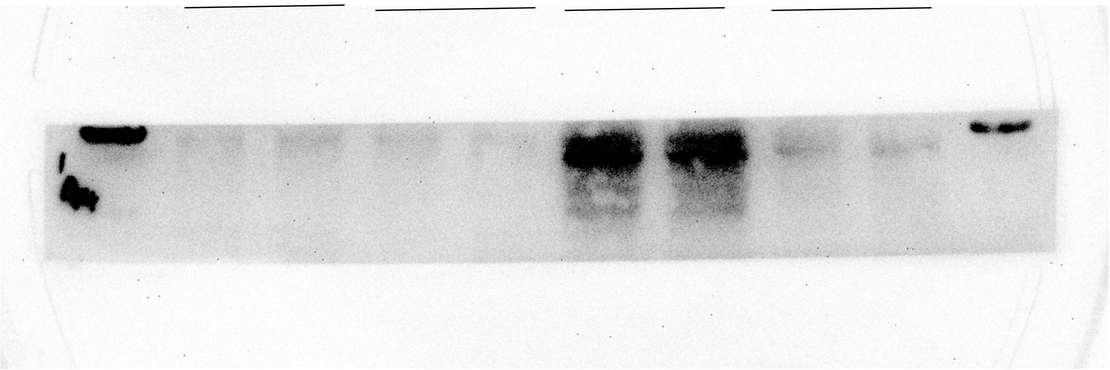

CHOP 30kD

Sham OE I/R OE+I/R

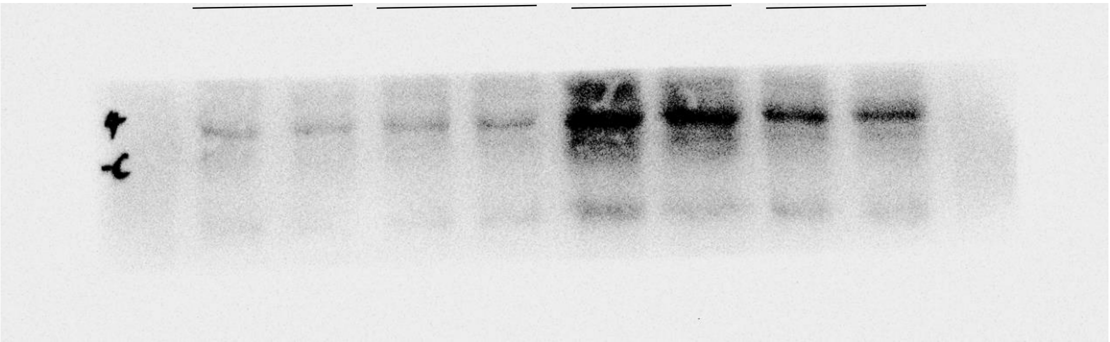

Cleaved-caspase3 17kD

Sham OE I/R OE+I/R

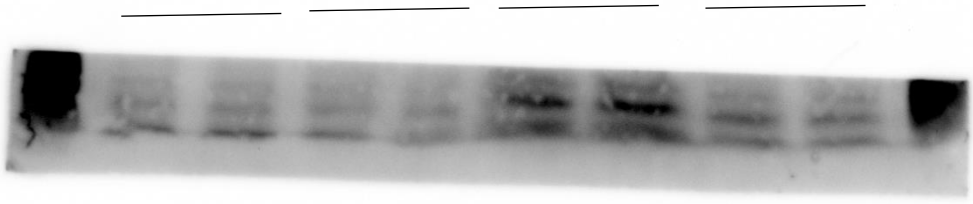

b-actin 42kD

Sham OE I/R OE+I/R

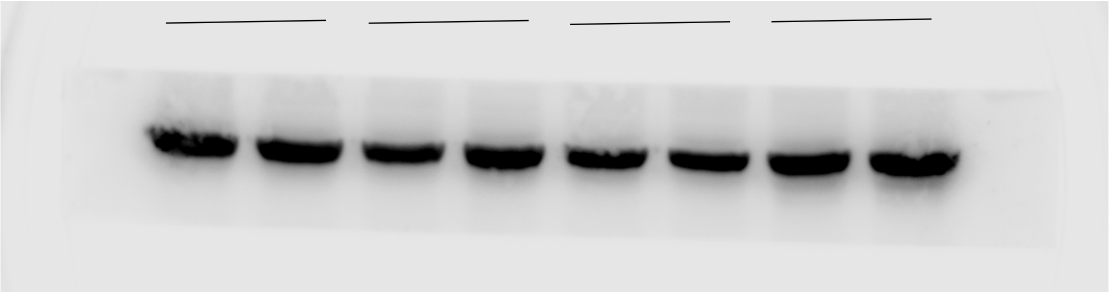

Fig.6

VDR 48kD

Control Pari TM TM+Pari

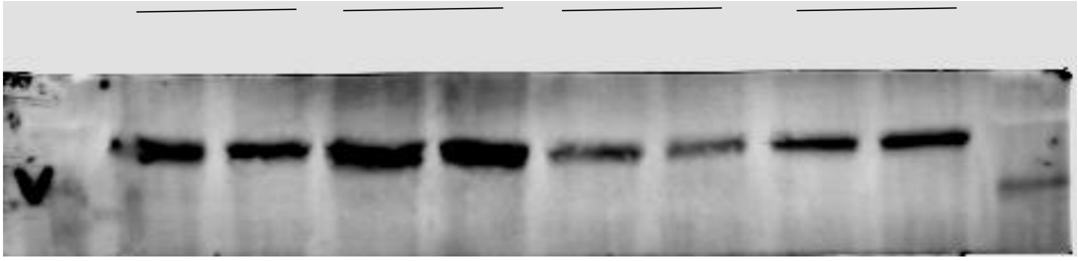

BiP 78kD

Control Pari TM TM+Pari

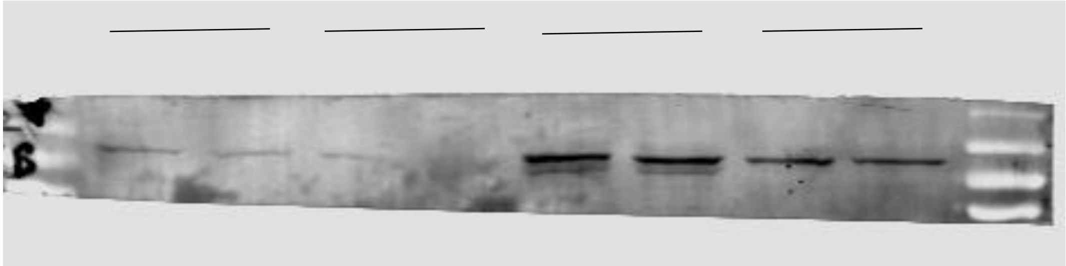

ATF4 50kD

Control Pari TM TM+Pari

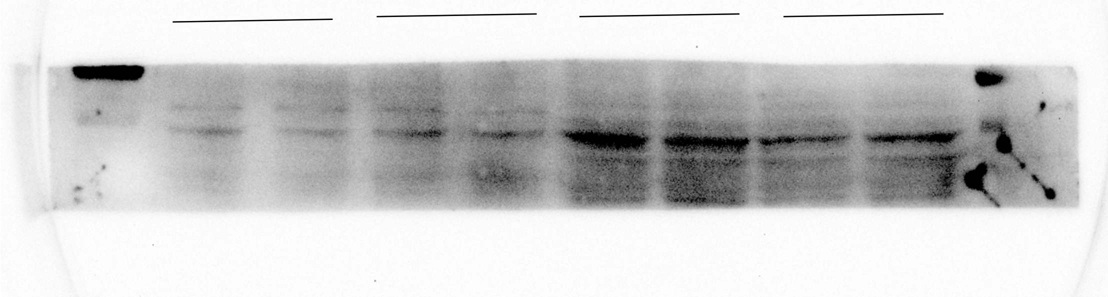

CHOP 30kD

Control Pari TM TM+Pari

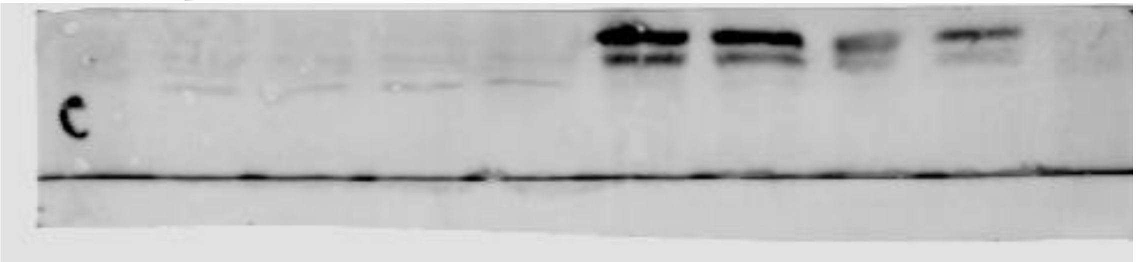

b-actin 42kD

Control Pari TM TM+Pari

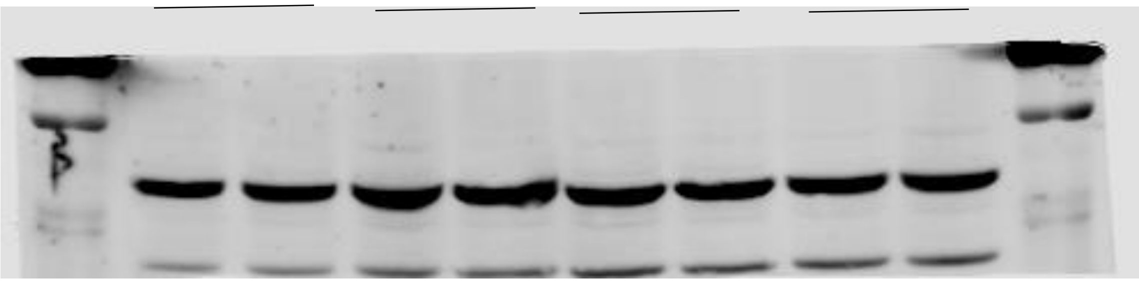

Fig.7

Fig.7 B

ATF4 50kD

NC ATF4-OE

Control TM TM+Pari Control TM TM+Pari

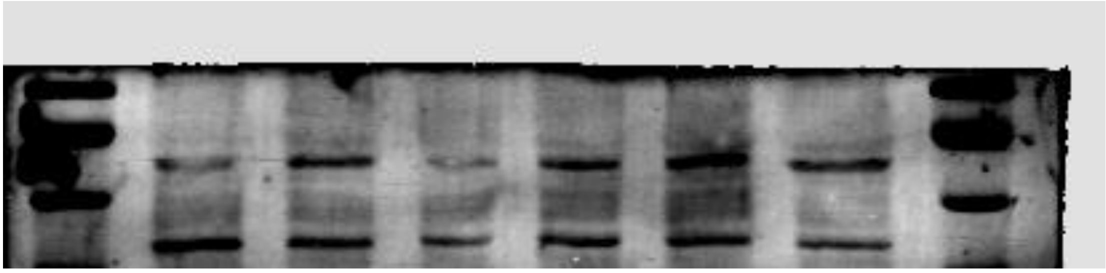

CHOP 30kD

NC ATF4-OE

Control TM TM+Pari Control TM TM+Pari

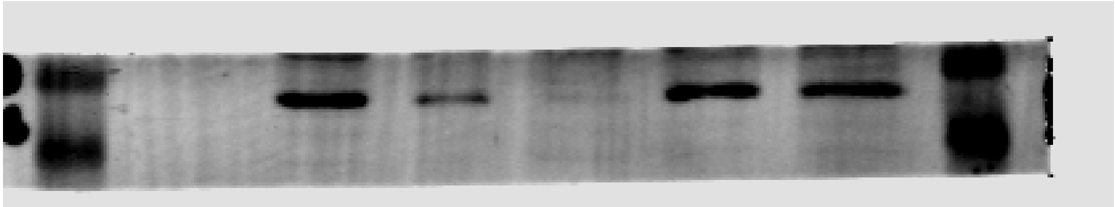

Cleaved-caspase3 17kD

NC ATF4-OE

Control TM TM+Pari Control TM TM+Pari

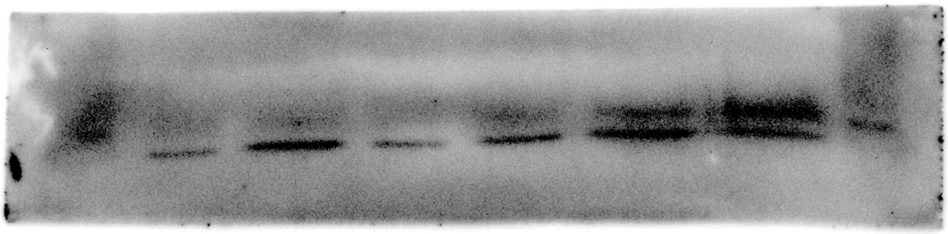

b-actin 42kD

NC ATF4-OE

Control TM TM+Pari Control TM TM+Pari

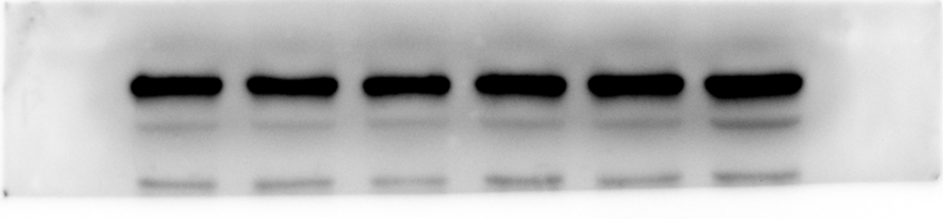

Fig.7 D

ATF4 50kD

NC			siATF4		
Control	TM	TM+Pari	Control	TM	TM+Pari

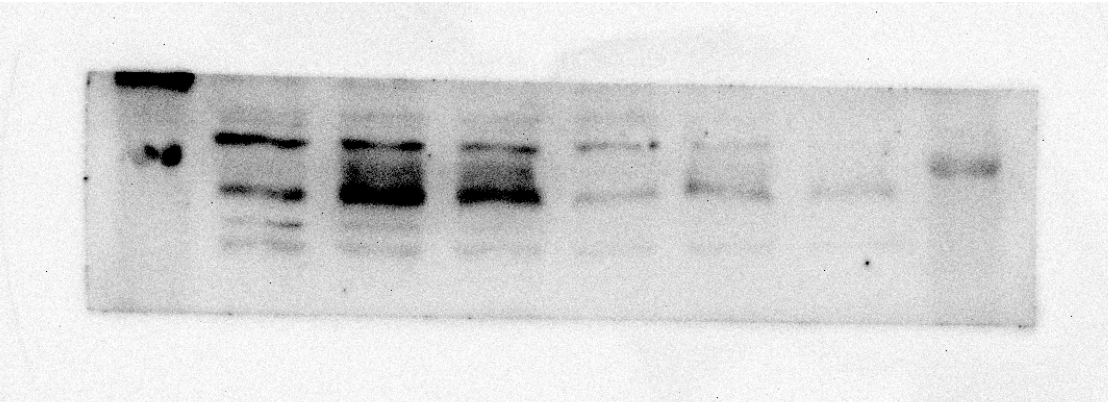

CHOP 30kD

NC			siATF4		
Control	TM	TM+Pari	Control	TM	TM+Pari

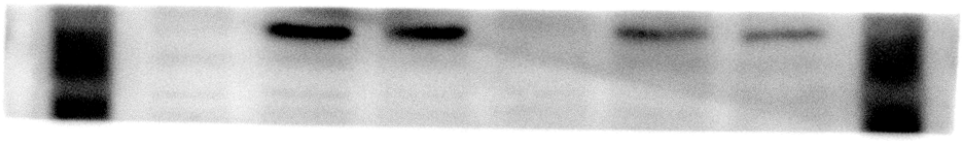

Cleaved-caspase3 17kD

NC			siATF4		
Control	TM	TM+Pari	Control	TM	TM+Pari

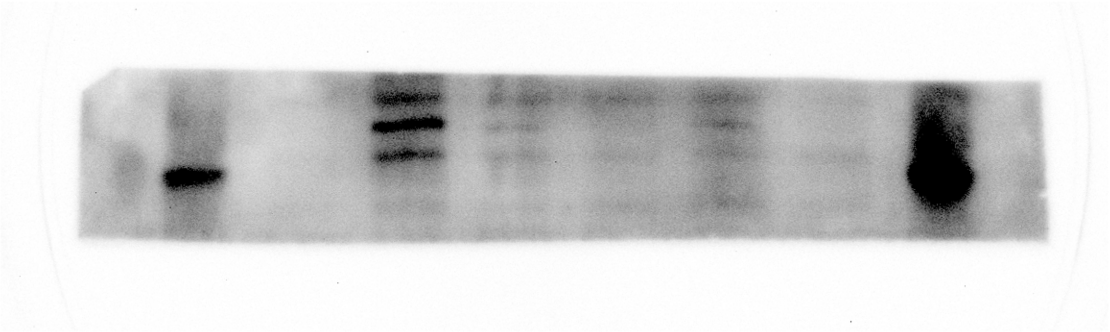

b-actin 42kD

NC			siATF4		
Control	TM	TM+Pari	Control	TM	TM+Pari

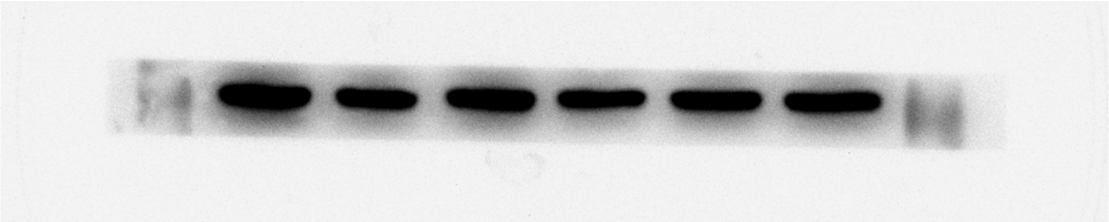

Fig.S1

BiP 78kD

WT KO TM KO+TM

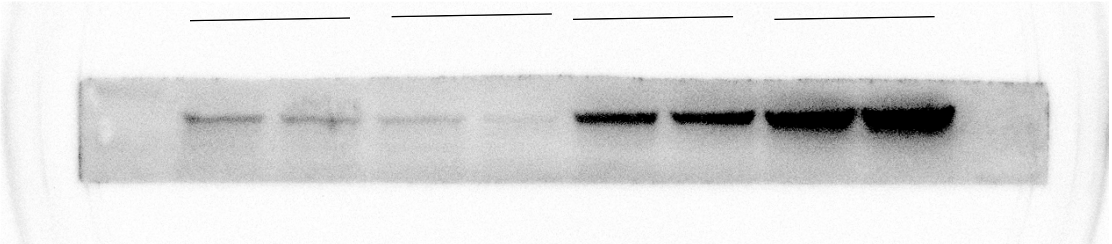

ATF4 50kD

WT KO TM KO+TM

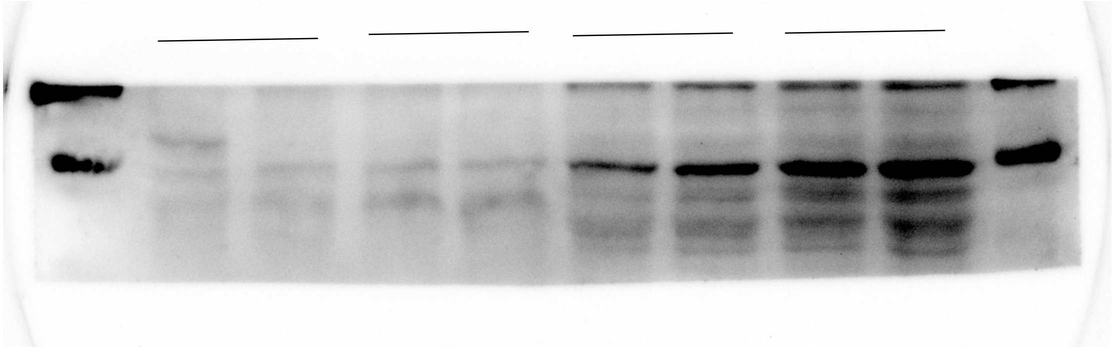

CHOP 30kD

WT KO TM KO+TM

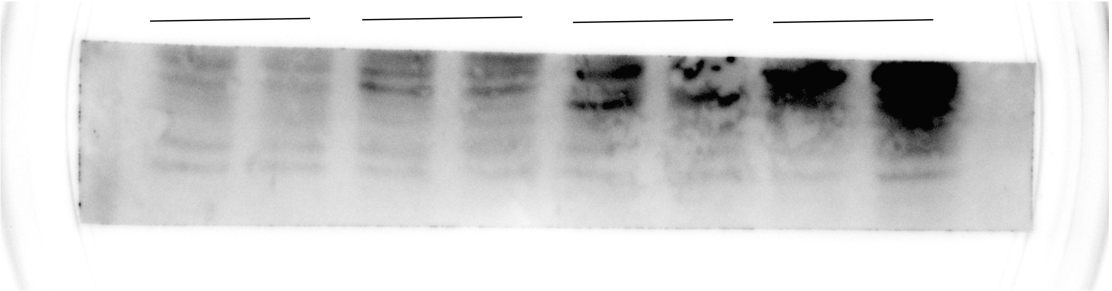

b-actin 42kD

WT KO TM KO+TM

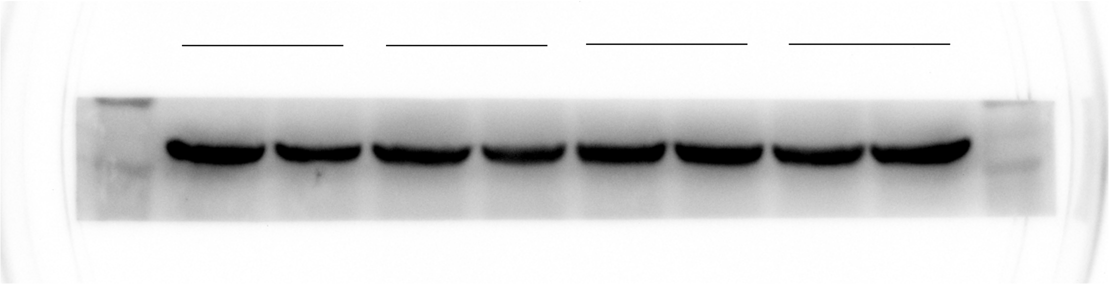

Supplement: Supplementary file 3 — Original western blots [file 41420_2023_1456_MOESM3_ESM.pdf]
